# Whole-genome sequence of *Kocuria rhizophila* 01AKB, an endophyte from the green macroalga *Halimeda* sp. in Kakaban Lake, Borneo, Indonesia

**DOI:** 10.1128/mra.00284-26

**Published:** 2026-05-06

**Authors:** Andri Frediansyah, Hayatun Nufus, Ajeng Kusumaningtyas Pramono, Abdul Rahman Siregar, Harald Gross

**Affiliations:** 1 Research Center for Food Technology and Processing, National Research and Innovation Agency (BRIN), Wonosari, Daerah Istimewa Yogyakarta, Indonesia; 2 Health Department of Ngawi Regency, Public Health Laboratory, Ngawi, East Java, Indonesia; 3 Research Center for Biomedical, National Research and Innovation Agency (BRIN), Cibinong, Jawa Barat, Indonesia; 4 Faculty of Biology, Gadjah Mada University 275023 , Yogyakarta, Indonesia; 5 Department of Pharmaceutical Biology, Pharmaceutical Institute, University of Tübingen 9188, Tübingen, Germany; California State University San Marcos, San Marcos, California, USA

**Keywords:** *Kocuria*, endophytes

## Abstract

*Kocuria rhizophila* 01AKB was isolated from the inner tissue of the green macroalga *Halimeda* sp. in the unique brackish water ecosystem of Kakaban Lake, Indonesia. Here, we report its complete genome sequence, which provides a foundation for understanding the ecological role and biotechnological potential of this endophytic bacterium.

## ANNOUNCEMENT

Kakaban Lake in Borneo, Indonesia, is a saline lake with a unique ecosystem, foremost due to its anchialine character and the massive presence of stingless jellyfish (1, 2). In a study of extremophilic bacteria inhabiting such environments, strain *Kocuria* sp. 01AKB was isolated in August 2022, as an endophyte from the green macroalga *Halimeda* sp. (2.1403°N, 118.5091°E). The macroalga was surface-sterilized with 70% ethanol (2 min) and 1% sodium hypochlorite (1 min), then rinsed three times with sterile seawater. The inner tissue was extracted under aseptic conditions, homogenized in sterile seawater, serially diluted (10⁻¹ to 10⁻⁵), and 100 µL aliquots were plated on Zobell marine agar (3, 4). Plates were incubated at 25°C for 48 h. Members of the genus *Kocuria* are known for their resilience in diverse environments and potential for bioactive compound synthesis (5). To elucidate its adaptive mechanisms and biosynthetic potential, we performed whole-genome sequencing and *in silico* analysis of strain 01AKB.

A 1 mL culture of strain 01AKB from a single colony grown in Zobell marine broth (3, 4) for 24 h at 25°C and 160 rpm was used for genomic DNA extraction. The cell pellet was treated with lysozyme, and DNA was purified using the Wizard Genomic DNA Purification Kit (Promega). DNA purity and concentration (116 ng/µL) were assessed via NanoDrop spectrophotometry and Qubit 3.0 Fluorometer, respectively. A sequencing library was prepared using the SQK-NBD114-24 kit (Oxford Nanopore Technologies) without additional shearing; size selection was not performed. Sequencing was performed on a MinION device with a FLO-PRO114M flow cell using MinKNOW v24.06.10.

Raw FASTQ files were generated at 10-min intervals using MinKNOW’s integrated basecaller (Guppy v6.4.6, high-accuracy model). Adapter and barcode trimming were performed downstream using Guppy v3.2.6. Quality control included filtering reads with quality scores below Q9. *De novo* genome assembly was conducted using the EPI2ME Labs wf-bacterial-genomes 56 Nextflow pipeline, which performed assembly with Flye v2.9.6 (6), polishing with Medaka v2.0.0, and contig reorientation with dnaapler v0.5.0. Genome annotation was performed with PGAP v6.10 (7, 8). For all software tools, default parameters were used unless otherwise specified. Table 1 summarizes the genomic information. Analysis of dDDH *d*_4_ and ANI values (Table 1) showed unambiguously in both cases that strain 01AKB represents a *Kocuria rhizophila* strain. As determined by BLAST analysis, the plasmid of 01AKB shared 99.97% identity with a plasmid from *Micrococcus luteus* UC81_27 (CP172001.1). Secondary metabolism analysis using antiSMASH v8 (9) (Table 1) unveiled several biosynthetic gene clusters with low similarity to known clusters, indicating potential for novel metabolite discovery.

**TABLE 1:** Sequencing metrics for *Kocuria rhizophila* 01AKB and bioinformatic analysis results

Statistic or characteristic	Data for strain 01AKB
Sequencing	
No. of reads	1,245,832
Subread *N*_50_ (bp)	13,204
Min./max. sequence length (bp)	1,000/130,526
Coverage (×)	330
Assembly	
No. of contigs	2
Contig 1 (chromosome) size (bp)	2,692,540
Contig 2 (plasmid) size (bp)	4,132
GC content (%) (chromosome/plasmid)	70.6/43.9
Estimated genome completeness (%)*^a^*	99
No. of protein-coding genes (chromosome/plasmid)	2,299/5
rRNAs	3, 3, 3 (5S, 16S, 23S)
tRNAs	47
WGS-based screening for biosynthetic gene clusters*^b^*	
Terpene	2
RiPP	1
Type III polyketide	1
Siderophore	1 (aerobactin-like)
Non-alpha poly-amino acids like e-polylysin	1
Betalactone	1
WGS-based taxonomy	
dDDH value *d*_4_ (%)*^c^* (closest type strain)	72.9 (*Kocuria rhizophila* TA68^T^)
ANI value (%)^*d*^ (closest type strain)	97.7 (*Kocuria rhizophila* TA68^T^)

^*a*^ Determined by BUSCO v5.8.2 analysis using the kocuria_odb12 data set.

^*b*^ Biosynthetic gene clusters coding for secondary metabolites identified using antiSMASH v8. The output files are publicly accessible on Zenodo (https://doi.org/10.5281/zenodo.19535087).

^*c*^ dDDH: digital DNA-DNA-hybridization, determined with TYGS.

^*d*^ ANI: average nucleotide identity, determined with autoMLST2.

## Data Availability

The whole-genome assembly of strain 01AKB is available at NCBI GenBank (accession number JBUXKX000000000), and raw sequencing reads have been deposited under accession number SRX32175518.
